# Internal limiting membrane peeling and gas tamponade for myopic foveoschisis: a systematic review and meta-analysis

**DOI:** 10.1186/s12886-017-0562-8

**Published:** 2017-09-08

**Authors:** Bo Meng, Lu Zhao, Yi Yin, Hongyang Li, Xiaolei Wang, Xiufen Yang, Ran You, Jialin Wang, Youjing Zhang, Hui Wang, Ran Du, Ningli Wang, Siyan Zhan, Yanling Wang

**Affiliations:** 1 Beijing Friendship Hospital, Capital Medical University, 95 Yong-an Road., Xi-Cheng District, Beijing, 100050 China; 2 Beijing Tongren Eye Center, Beijing Tongren Hospital, Capital Medical University, Beijing, China; 3 Department of Epidemiology and Biostatistics, School of Public Health, Peking University Health Science Centre, Beijing, China

**Keywords:** Internal limiting membrane peeling, Tamponade, Myopic foveoschisis, Systematic review

## Abstract

**Background:**

Myopic foveoschisis (MF) is among the leading causes of visual loss in high myopia. However, it remains controversial whether internal limiting membrane (ILM) peeling or gas tamponade is necessary treatment option for MF.

**Methods:**

PubMed, EMBASE, CBM, CNKI, WANFANG DATA and VIP databases were systematically reviewed. Outcome indicators were myopic foveoschisis resolution rate, visual acuity improvement and postoperative complications.

**Results:**

Nine studies that included 239 eyes were selected. The proportion of resolution of foveoschisis was higher in ILM peeling group than non-ILM peeling group (OR = 2.15, 95% CI: 1.06–4.35; *P* = 0.03). The proportion of postoperative complications was higher in Tamponade group than non-Tamponade group (OR = 10.81, 95% CI: 1.26–93.02; P = 0.03). However, the proportion of visual acuity improvement (OR = 1.63, 95% CI: 0.56–4.80; *P* = 0.37) between ILM peeling group and non-ILM peeling group and the proportion of resolution of foveoschisis (OR = 1.80, 95% CI: 0.76–4.28; *P* = 0.18) between Tamponade group and non-Tamponade group were similar.

**Conclusions:**

Vitrectomy with internal limiting membrane peeling could contribute to better resolution of myopic foveoschisis than non-peeling, however it does not significantly influence the proportion of visual acuity improvement and postoperative complications. Vitrectomy with gas tamponade is associated with more complications than non-tamponade and does not significantly influence the proportion of visual acuity improvement and resolution of myopic foveoschisis.

**Electronic supplementary material:**

The online version of this article (10.1186/s12886-017-0562-8) contains supplementary material, which is available to authorized users.

## Background

Myopic foveoschisis (MF) is among the leading causes of visual loss in high myopia. It has been named shallow detachment of the macula, foveal retinoschisis, macular retinoschisis or posterior retinoschisis [1]. The optical coherence tomography (OCT) presentation of retinoschisis was firstly described in detail using time domain OCT in 1999 [2]. Since then, OCT scanning has become the predominant tool for the diagnosis of MF because of its reliability. The prevalence of MF is reported up to 34% [2–5]. However, the current prevalence figure of MF may indicate an underestimation not only in the whole population but also in stratified population based on age or refractive error because of the absence of uniformly adopted diagnostic criteria and large-scale studies exploring spectral domain optical coherence tomography (SD-OCT) for MF [6].

The pathogenesis of MF still remains unclear. Different factors are related to the occurrence and development of MF [6]. Anomalous or incomplete posterior vitreous detachment as well as contraction of attached cortical vitreous are considered to be the major factors. And other factors, for instance, excessive rigidity or poor elasticity of the internal limiting membrane (ILM), progressive posterior staphyloma, and stiffness of retinal vessels might play a part in the pathogenesis of MF as well [2, 6–11].

Vitrectomy has been proved to be an effective treatment for MF in many studies [11–18]. However, it remains controversial whether ILM peeling or gas tamponade is necessary treatment option for MF, and a consensus of the validity of this topic has not been reached [8, 19–26]. So we carried out a meta-analysis to evaluate the evidence available for the validity and safety of the following treatments for MF: vitrectomy with ILM peeling versus vitrectomy without ILM peeling, and vitrectomy with gas tamponade versus vitrectomy without gas tamponade. Our primary outcome to determine efficacy was resolution of macular hole. We also evaluated visual acuity as a secondary efficacy outcome.

## Methods

We performed a systematic review and a meta-analysis according to the recommendations of the Cochrane Handbook and reported according to the PRISMA Statement for meta-analyses and systematic reviews [27].

### Search strategy

We conducted a literature search to tell all the studies relevant that contrasted outcomes of vitrectomy with or without ILM peeling/gas tamponade for MF. PubMed, EMBASE, CBM, CNKI, WANFANG DATA and VIP databases were retrieved with no language restrictions from the inception to December 2016. The selected key words were used as free words, truncations and subject morphology. Detailed search strategy was shown in Additional file 1. We manually searched the reference lists of all retrieved articles for potentially eligible articles.

### Inclusion and exclusion criteria

All publications were screened by two people according to predefined selection criteria. Any disagreement was discussed by the two people and resolved. Inclusion criteria were as follows: (1) randomized or nonrandomized studies that evaluated proportion of resolution of MF, visual acuity improvement and postoperative complications after vitrectomy with ILM peeling versus vitrectomy without ILM peeling, and that after vitrectomy with gas tamponade versus vitrectomy without gas tamponade for MF; (2) refered to no less than one of the outcome indicators mentioned above; (3) if a study was reported in duplicate, the version with the most comprehensive content was included in this analysis.

The following listed were exclusion criteria: (1) non-comparative studies, single-arm studies, animal studies, case reports; (2) abstracts, letters, editorials and conference proceedings without original data or if from the published results, it was impossible to obtain proper data; (3) studies included cases with macular holes or retinal detachment.

### Data extraction

The extraction of data from each study was performed by two reviewers independently. Any disagreement was discussed by the two reviewers and resolved. The extracted information included year of publication, first author, study location, design, operation mode, follow-up time, number of eyes, age of patients, refractive errors, axial length.

### Assessment of study quality

Bias risk assessment tool is recommended in the Cochrane Handbook [28]. It has clear structure and is easy to use. In this meta-analysis, two people used this tool for assessment of study quality. Any disagreement was discussed by the two people and resolved. The following items were included: (1) allocation concealment (selection bias); (2) generation of random sequence (selection bias); (3) outcome assessment blinding (detection bias); (4) participants and personnel blinding (performance bias); (5) incomplete data (attrition bias); (6) reporting selectivity(reporting bias); (7) other bias.

### Outcome indicators of interest

The following outcome indicators were used to compare ILM peeling group and non-ILM peeling group, and to compare Tamponade group and non-Tamponade group. (1) Data of efficacy, includes the proportion of resolution of MF and the proportion of visual acuity improvement; (2) data of safety, includes the proportion of postoperative complications such as recurrence, macular hole, retinal detachment, hemorrhage, transient rise of intraocular pressure and cataract.

### Statistical analysis

Data analyses were conducted using RevMan 5.1. We analyzed dichotomous variables by means of estimation of ORand 95% CI. Heterogeneity was assessed by calculating the I^2^ and performing the chi-square test (to assess the *P* value) with I^2^ > 50% and *P* < 0.05 suggesting significant heterogeneity. We applied fixed effects model if there was no apparent heterogeneity. However, if there was any heterogeneity, we would use random effects model for meta-analysis. The funnel plot was applied for evaluation of publication bias.

## Results

### Selection of studies

**Fig. 1:**
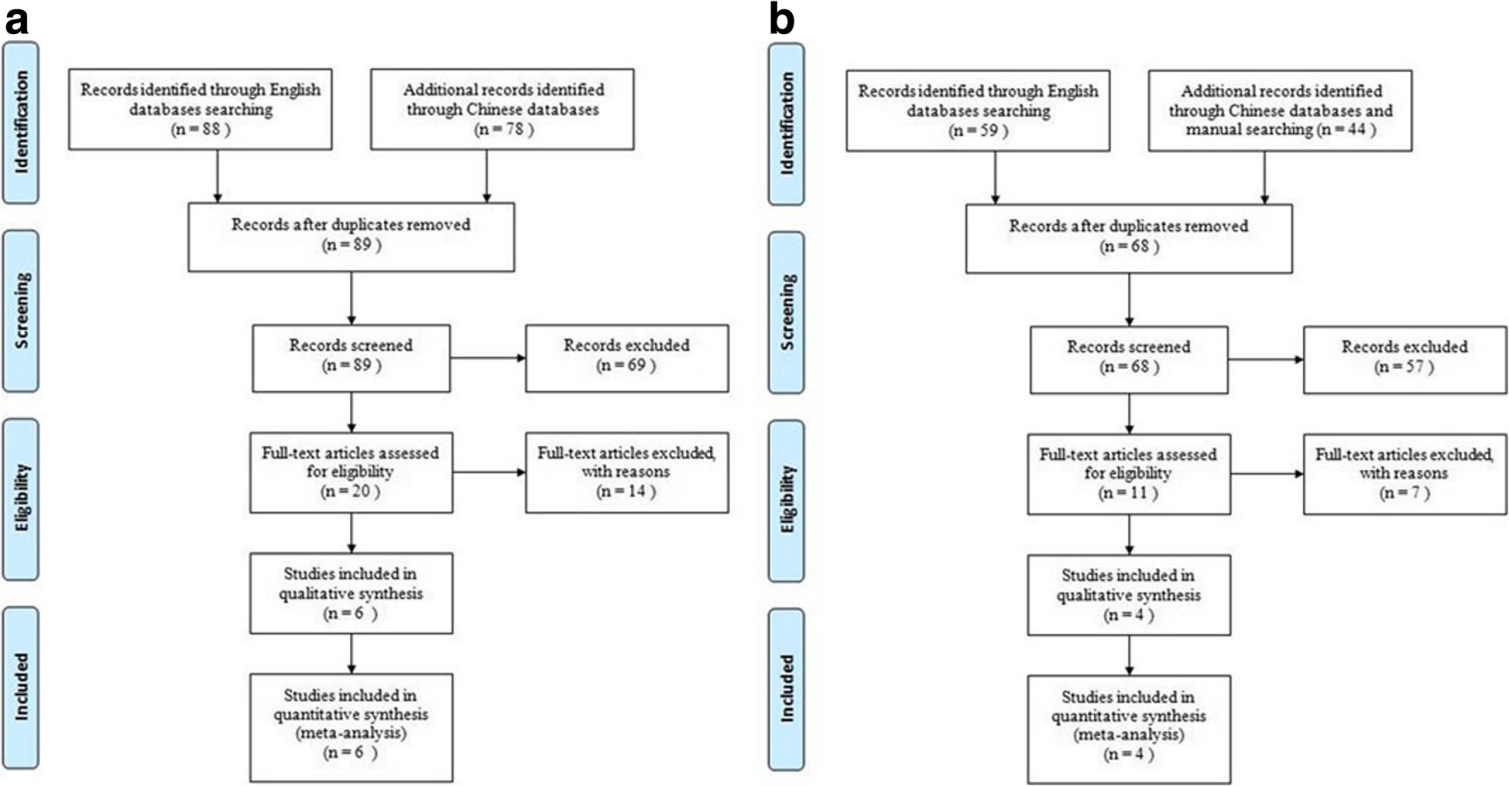
**a** Flowchart of selection process in the comparison of ILM peeling group and non-ILM peeling group. **b** Flowchart of selection process in the comparison of Tamponade group and non-Tamponade group

Initially, a total of 269 literature were retrieved. But the majority of these studies were not fit for this meta-analysis because they were non-comparative studies, single-arm studies, duplicates studies or case reports having noting to do with oursubject. After looked through all the titles, abstracts as well as full text, 259 articles were cut out in accordance with selection criteria and finally altogether 9 studies [8, 19–26] were included for our meta-analysis (Fig. 1).

### Characteristics of the included studies

In total, 9 studies that included 239 eyes were included. One study was prospective study, one was retrospective study, and the other studies were considered randomized controlled studies. Among them, 6 studies, [21–26] 186 eyes (86 eyes in ILM peeling group, 100 eyes in non-ILM peeling group) were included in the meta-analysis of vitrectomy with ILM peeling versus that without ILM peeling for the treatment of MF. And 4 studies, [8, 19, 20, 24] 109 eyes (53 eyes in Tamponade group, 56 eyes in non-Tamponade group) were included in the meta-analysis of vitrectomy with gas tamponade versus that without gas tamponade. In Table 1 and Table 2 were the characteristics of these studies summarized. The baseline characteristics of each included study, for instance, age, axial length and refractive error turned out to be comparative between two compared groups. In Additional file 2 were the types of outcomes measured such as visual acuity, macular hole resolution, complications.

**Table 1:** Characteristics of included studies in the comparison of ILM peeling group and non-ILM peeling group

		Study	Study		Follow-up	ILM Peeling				Non-ILM Peeling			
Author	Year	Location	Design	Operation Mode	Time(m)	Eyes	Age(y)	Refractive Errors (D)	Axis(mm)	Eyes	Age(y)	Refractive Errors (D)	Axis(mm)
Li et al. [22]	2007	China	PS	PPV + tamponade + ILM Peeling; PPV + tamponade	>3	4	NR	−17.5 ± 4.75	30.27 ± 2.13	7	NR	−17.2 ± 5.5	30.12 ± 3.28
Song et al. [24]^①^	2011	China	RCT	PPV + ILM Peeling; PPV	9	10	NR	NR	NR	24	NR	NR	NR
Song et al. [24]^②^	2011	China	RCT	PPV + tamponade + ILM Peeling; PPV + tamponade	9	13	NR	NR	NR	16	NR	NR	NR
Xu et al. [26]	2011	China	RCT	PPV + tamponade + ILM Peeling; PPV + tamponade	6 ~ 14	14	57 ± 6.9	−12.4 ± 2.6	29.1 ± 3.1	15	59 ± 8.7	−11.7 ± 2.8	28.3 ± 2.3
Cai et al. [21]	2011	China	RCT	PPV + ILM Peeling; PPV	3	14	35 ~ 79	−14 ~ −6	26.31 ~ 33.12	11	35 ~ 79	−14 ~ −6	26.31 ~ 33.12
Liu et al. [23]	2014	China	RCT	PPV + tamponade + ILM Peeling; PPV + tamponade	2	16	46.63 ± 7.54	−14.81 ± 4.53	30.8 ± 2.56	14	46.14 ± 8.44	−14.93 ± 4.43	31.08 ± 2.46
Wang et al. [25]	2014	China	RCT	PPV + tamponade + ILM Peeling; PPV + tamponade	6 ~ 11	15	50.4 ± 7.2	−9.93 ± 2.36	30.17 ± 1.28	13	53.15 ± 6	−10.31 ± 2.26	29.73 ± 1.3

*PS* prospective study, *RCT* randomized control trial, *PPV* pars plana vitrectomy, *NR* not reported, Song et al.^①^, Song et al.^②^: two sets of data in the study of Song et al

**Table 2:** Characteristics of included studies in the comparison of Tamponade group and non-Tamponade group

		Study	Study		Follow-up	Tamponade				Non-Tamponade			
Author	Year	Location	Design	Operation Mode	Time(m)	Eyes	Age(y)	Refractive Errors (D)	Axis(mm)	Eyes	Age(y)	Refractive Errors (D)	Axis(mm)
Zhang et al. [19]	2010	China	RCT	PPV + tamponade; PPV	9	16	54.8 ± 13.3	−20.3 ~ −13.6	30.2 ± 1.6	24	52.7 ± 11.9	−19.7 ~ −14.1	30.8 ± 2
Song et al. [24]	2011	China	RCT	PPV + ILM Peeling + tamponade; PPV + ILM Peeling	9	13	NR	NR	NR	10	NR	NR	NR
Kim et al. [8]	2012	Korea	RS	PPV + ILM Peeling + tamponade; PPV + ILM Peeling	12	9	61.78 ± 8.98	−18.21 ± 4.36	29.31 ± 1.1	8	62.13 ± 10.1	−14.21 ± 3.1	30.24 ± 1.55
Gui et al. [20]	2015	China	RCT	PPV + ILM Peeling + tamponade; PPV + ILM Peeling	6	15	42.56 ± 3.74	−11.54 ± 5.26	27.06 ± 3.24	14	42.56 ± 3.74	−11.54 ± 5.26	27.06 ± 3.24

*RS* retrospective study, *RCT* randomized control trial, *PPV* pars plana vitrectomy, *NR* not reported

### Assessment of quality

The quality assessment of the studies incorporated was described comprehensively in Additional file 3.

### Resolution of myopic foveoschisis

**Fig. 2:**
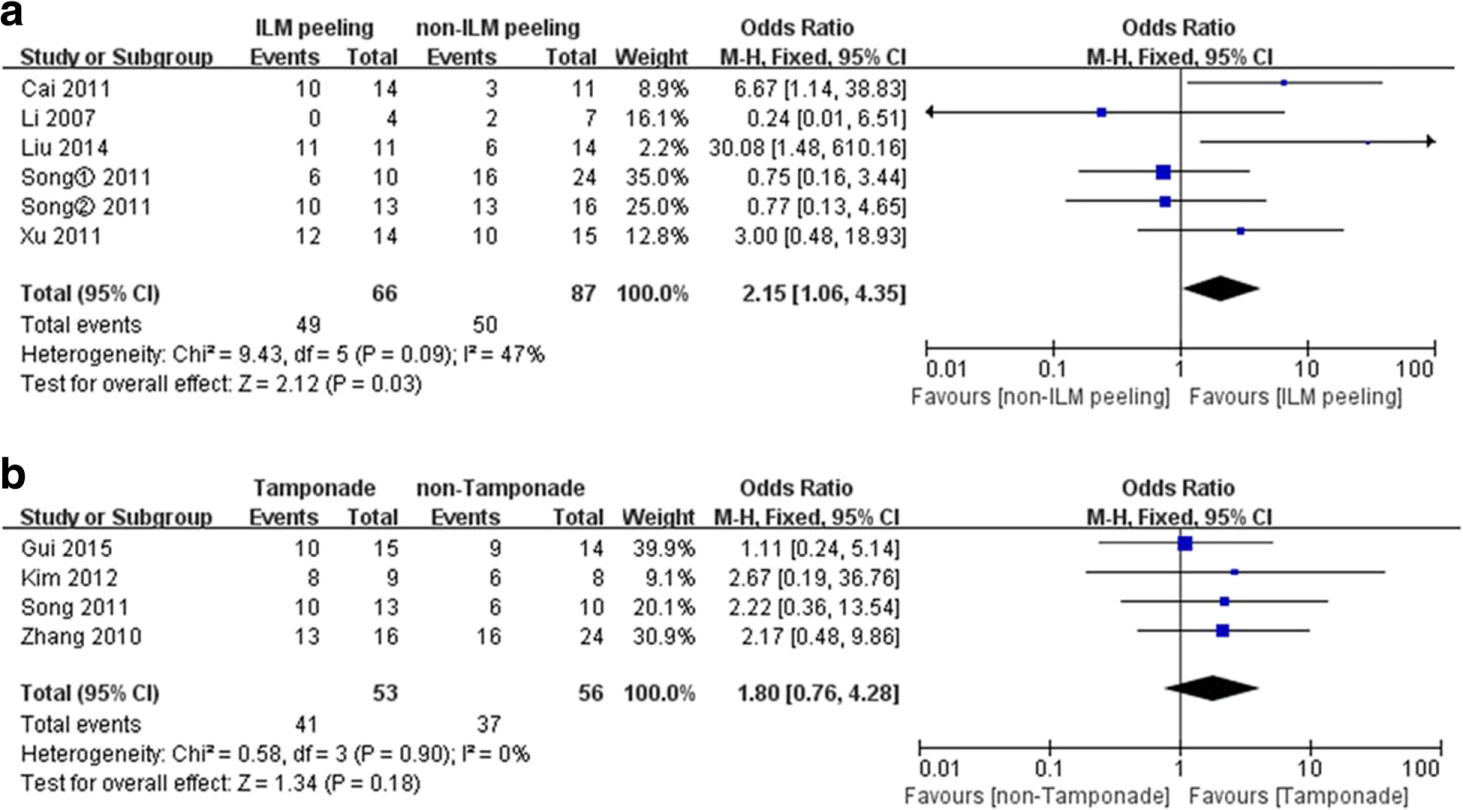
**a** A forest plot showing the proportion of resolution of MF between ILM peeling group and non-ILM peeling group. **b** A forest plot showing the proportion of resolution of MF between Tamponade group and non-Tamponade group. Song^①^, Song^②^: two sets of data in the study of Song et al

The proportion of resolution of MF was reported in 5 studies including 153 eyes in ILM peeling group compared with non-ILM peeling group. No statistical heterogeneity was found between the studies (*P* = 0.09, I^2^ = 47%). It turned out by fixed effects model that the proportion of resolution of MF was higher in ILM peeling group than that in non-ILM peeling group (OR = 2.15, 95% CI: 1.06–4.35; *P* < 0.05) (Fig. 2a). However, patients undergoing vitrectomy with gas tamponade experienced a similar resolution of MF with those undergoing vitrectomy with no tamponade (4 studies including 109 eyes). No significant heterogeneity was found (*P* = 0.90, I^2^ = 0%). It turned out by fixed effects model that the regard of the two groups was of no significant differences (OR = 1.80, 95% CI: 0.76–4.28; *P* > 0.05) (Fig. 2b).

### Visual acuity improvement

**Fig. 3:**
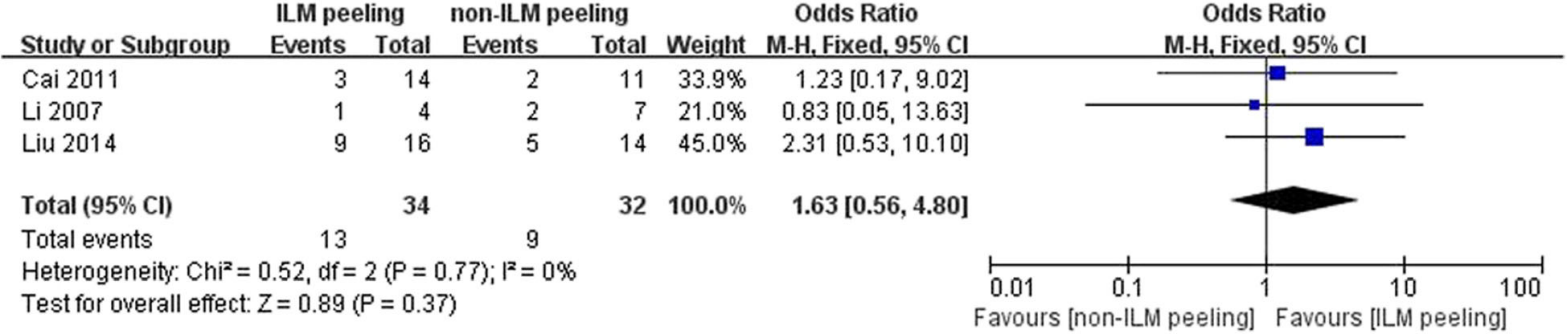
A forest plot showing the proportion of visual acuity improvement between ILM peeling group and non-ILM peeling group

Visual acuity was displayed in decimal. If visual acuity was displayed in logMAR, each 0.1 logMAR unit represent 1 line. And visual acuity which improved more than 1 lines was considered effective [29]. The consolidated data from 3 studies containing 94 eyes indicated that the ILM peeling group had a similar visual acuity improvement proportion than the non-ILM peeling group (OR = 1.63, 95% CI: 0.56–4.80; *P* > 0.05) and no statistically significant heterogeneity was found between the two groups (*P* = 0.77, I^2^ = 0%) (Fig. 3). However, the meta-analysis of vitrectomy with gas tamponade versus vitrectomy with no tamponade could not be achieved because all the patients had visual acuity improvement in both two groups in two [19, 20] among three included studies [8, 19, 20].

### Postoperative complications

**Fig. 4:**
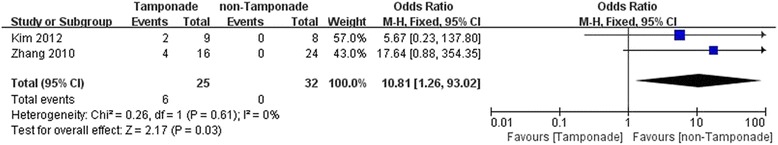
A forest plot showing the proportion of postoperative complications between Tamponade group and non-Tamponade group

Four studies [21–23, 26] reported postoperative complications such as recurrence, hemorrhage, macular hole, retinal detachment, cataract and transient rise of intraocular pressure in the topic of vitrectomy with ILM peeling versus vitrectomy without ILM peeling. However, the meta-analysis could not be achieved because three studies [21–23] had no complications in both two groups. Two studies fitted into the meta-analysis of vitrectomy with gas tamponade versus vitrectomy without tamponade. No statistically significant heterogeneity was found between the trials (*P* = 0.61, I^2^ = 0%). It is suggested by a fixed effects model that the postoperative complications proportion was higher in the group of vitrectomy with gas tamponade than vitrectomy with no tamponade (OR = 10.81, 95% CI: 1.26–93.02; *P* < 0.05) (Fig. 4).

### Publication bias

**Fig. 5:**
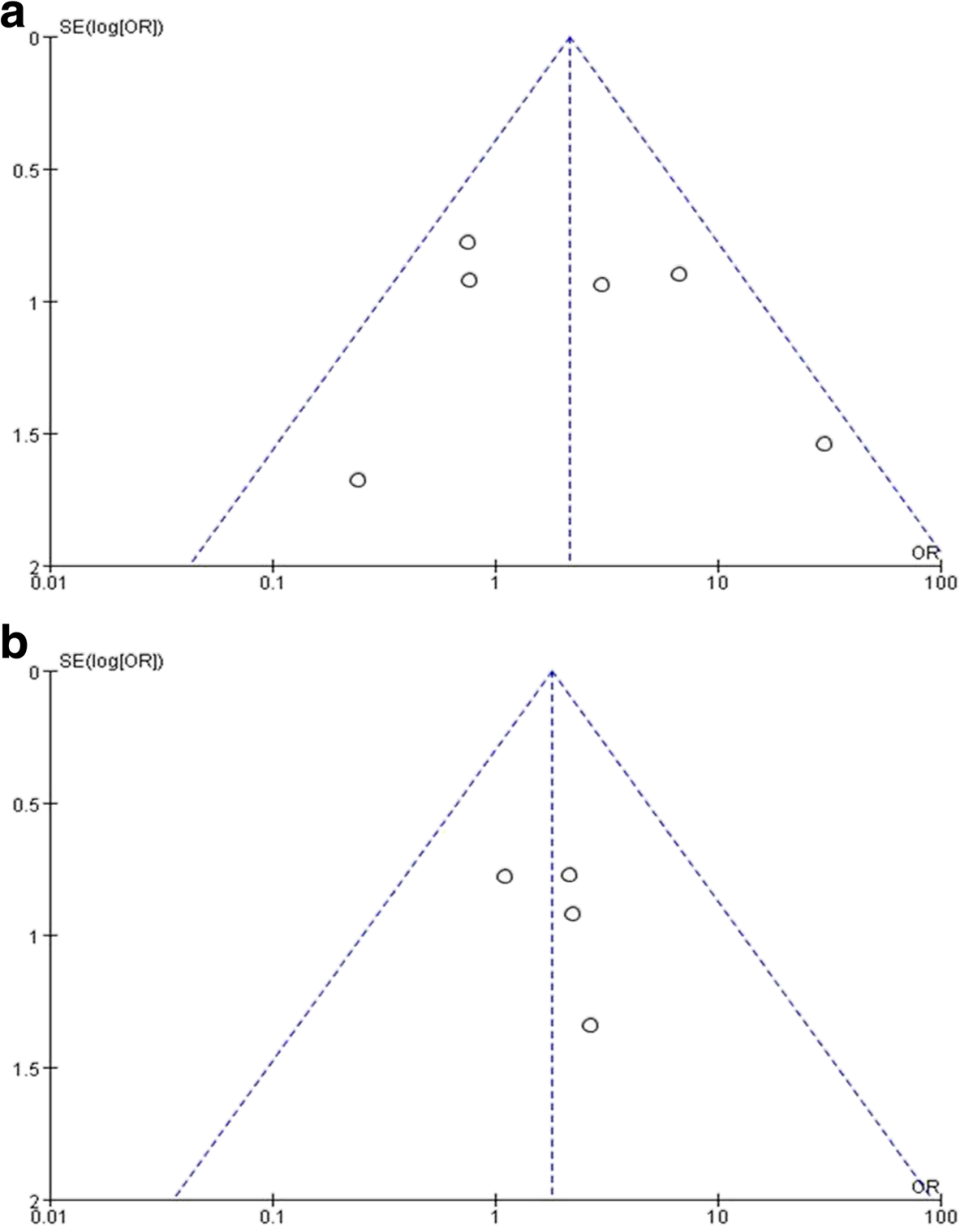
Funnel plots of the included studies comparing the proportion of resolution of MF of retinal reattachment showing no significant publication bias. **a** ILM peeling group versus non-ILM peeling group. **b** Tamponade group versus non-Tamponade group. SE = standard error, OR = odds ratio

The proportion of resolution of MF revealed symmetry on a funnel plot. This suggested that there was no publication bias (Fig. 5).

## Discussion

Vitrectomy is considered as a helpful therapeutic method for MF [12, 18, 30]. However, there remains debate on this operation process, with regard to the essentiality of gas tamponade or ILM peeling. It has been proved that vitrectomy with ILM peeling resulted in the resolution of MF and postoperative vision enhancement in more than 70% of the patients within 6 months [8, 9, 15–17, 31, 32]. But many other researchers considered that vitrectomy with no ILM peeling may also lead to favorable anatomic and functional outcomes, which was equivalent to the operation mode of ILM peeling only [11, 14, 18]. As a therapeutic method for MF, gas tamponade has been used in clinical, and it can induce retinal reposition via pushing back the retina. Yet further study is needed to confirm whether gas tamponade is absolutely necessary in the treatment prescription of MF [9, 31].

Our meta-analysis summarizes the evidence available for the validity and safety of ILM peeling group versus that of non-ILM peeling group, and the validity and safety of Tamponade group versus that of non-Tamponade group for the treatment of MF. The results indicate that ILM peeling could contribute to better resolution of foveoschisis outcome but it does not significantly improve postoperative visual acuity than non-ILM peeling in patients with MF. Though there are few complications in ILM peeling group and non-ILM peeling group, the proportion of postoperative complications is proximate. This study also shows that vitrectomy with gas tamponade does not significantly improve resolution of MF and even has higher postoperative complications proportion than vitrectomy without gas tamponade. Though there is improvement of visual acuity in both two groups, the proportion of this outcome is similar between the two groups.

It has been reported that the separation of retinal layers in MF might be the result of inward pull, which was brought about by the relative resistance to a traction of the progressive ectasia of sclera and the inner retinal structures [2, 6, 33–36]. Histologic study of excised ILM from the eyes with MF found that fibroblast proliferation and the existence of cell debris or collagen fibres on the inner surface of peeled ILM was postulated to produce effects in the pathogenesis of MF [35]. And Wu et al. found that chorioretinal atrophy, the status of vitreoretinal interface and axial length more than 31 mm were related to the existence of MF in high myopia [37]. However, axial length in high myopia tends to be stable before 30-year-old, yet the occurrence of MF is much later. Therefore, it is more credible that the formation of MF are mainly owing to abnormal detachment of posterior vitreous as well as shrinkage of posterior cortical vitreous [33–35, 38].

With or without ILM peeling, pars plana vitrectomy (PPV) has been extensively accepted as the treatment standard for MF in highly myopic eyes, so ILM peeling may be not so important under this circumstance. However, it ensures complete elimination of tractional factors, for instance, premacular glial cells and vitreous cortex plaques on the ILM’s surface. Besides, via reducing the rigidity brought about by ILM, this can lead to better conformation of retina to staphyloma [12, 15, 39]. Nevertheless, complications such as macular hole, macular haemorrhage, ocular hypertension or retinal breaks can occur, especially in pathological myopia because of retinal thinning. Furthermore, vision enhancement is not always achieved after the reattachment of macula. No significant difference was found concerning the proportion of postoperative complications between ILM peeling group and non-ILM peeling group. We thought that the application of the dye when ILM peeling or the improvement of surgical skills avoided the adverse consequences of ILM peeling. Besides, in our study, ILM peeling group could have better resolution of foveoschisis outcome but patients in this group did not have significantly improved postoperative visual acuity than non-ILM peeling group. This gives us a hint that anatomical repositioning of the foveoschisis should been considerd before the damage of photoreceptor to achieve better recovery of function.

Some studies used gas in the final stage of vitrectomy to flatten the retina and weaken the vitreoretinal traction. It is not totally clear how this filling could better anatomical restoration in MF, though a few factors may be correlated with this mechanism. Above all, the gas is able to reduce the detachment by making retinal pigment epithelium (RPE) and retina together. Once subretinal fluid is expelled out of the submacular area, healthier RPE cells can pump it out with ease [40–42]. Other researchers consider that gas can generate a relative dry environment in macular lutea, which may have some effect on promoting the reabsorption of the resident fluid. And this conversely benefits the transport of oxygen and metabolites to external layer of the retina [11, 40]. However, the mechanical action of the gas could sustain only for one or two months. Nevertheless, the resolution of foveoschisis needs more than this time in many cases, which increases the difficulty in understanding the accurate mechanism of gas tamponade [8]. It has been reported in a few studies that the gas tamponade could accelerate anatomical resolution of MF [7, 8]. But Kumagai et al. reported that although there was a tendence of preferable visual outcome for eyes with gas tamponade, gas tamponade was not significantly related to final best corrected visual acuity [9]. In the current meta-analysis, gas tamponade does not improve the proportion of resolution of MF and the visual acuity, and even it has higher postoperative complication proportion than non-tamponade. This may be associated with the toxicity of the filler to the retina.

Our meta-analysis has several limitations which should be given a caution. Firstly, all the studies available for the meta-analysis had short-term follow-up periods and included small number of eyes or studies especially in the topic of gas tamponade. This may have lower representation and introduce observer bias. Secondly, the existing studies were based on Asians, so the ethnic background may affect the extrapolation of our results. Further prospective and randomized controlled clinical trials from other parts of the world especially Europe and the United States are necessary for deciding the optimal operation mode for the treatment of MF. Thirdly, successful surgical procedure depends on individual experiences of the surgeons. Therefore, the efficacy and safety outcomes such as the proportion of resolution of myopic foveoschisis, visual acuity improvement and postoperative complications might be affected to some extent. In addition, some surgeons might deal the eyes which had higher refractive error and/or longer symptom duration with simple operation such as no ILM peeling when doing vitrectomy to avoid postoperative complications or no gas tamponade to avoid toxicity to the retina. This may introduce evident selection bias. Finally, although the funnel plot demonstrated no publication bias and fixed or random effects model was used to test heterogeneity in our meta-analysis, it should be noticed that publication bias and heterogeneity usually presented due to few studies.

## Conclusions

In conclusion, the meta-analysis shows that vitrectomy with ILM peeling could contribute to better resolution of foveoschisis as compared to vitrectomy without internal limiting membrane peeling, although no significant differences were found in the outcomes of the visual acuity after operation as well as complications. In addition, our study finds that vitrectomy with gas tamponade may cause more complications as compared to vitrectomy without tamponade, although no significant differences were found in the outcomes of the visual acuity after operation as well as resolution of foveoschisis. However, our findings need to be confirmed by more randomized and prospective studies with longer duration of follow-up.

## Additional files

Additional file 1: 1. Searching strategy in pubmed for the comparison of ILM peeling group & non-ILM peeling group. 2. Searching strategy in pubmed for the comparison of Tamponade group & non-Tamponade group. (DOC 30 kb)

Additional file 2: 1. Outcome indicators of included studies in the comparison of ILM peeling group & non-ILM peeling group. MF, myopic foveoschisis; NR, not reported; Song et al.①, Song et al.②: two sets of data in the study of Song et al. 2. Outcome indicators of included studies in the comparison of Tamponade group & non-Tamponade group. MF, myopic foveoschisis; NR, not reported. (DOC 79 kb)

Additional file 3: Figure S1. a. Graph for risk of bias: All included studies are reviewed concerning the authors’ judgements on each bias risk item displayed as percentagesin the comparison of ILM peeling group & non-ILM peeling group. b. Summary for risk of bias: All included studies are reviewed concerning the authors’ judgements on each bias risk item in the comparison of ILM peeling group & non-ILM peeling group. **Figure S2.** a. Graph for risk of bias: All included studies are reviewed concerning the authors’ judgements on each bias risk itemdisplayed as percentages in the comparison of Tamponade group & non-Tamponade group. b. Summary for risk of bias: All included studies are reviewed concerning the authors’ judgements on each bias risk item in the comparison of Tamponade group & non-Tamponade group. (DOCX 254 kb)

## Abbreviations

95% CI: 95% Confidence Interval
CBM: Chinese Biomedical Literature Database
CNKI: China National Knowledge Infrastructure
ILM: Internal Limiting Membrane
MF: Myopic Foveoschisis
OCT: Optical Coherence Tomography
OR: Odds Ratio
PPV: Pars Plana Vitrectomy
SD-OCT: Spectral Domain Optical Coherence Tomography
