# Supplementary material for: Internal limiting membrane peeling and gas tamponade for myopic foveoschisis: a systematic review and meta-analysis
Source: BMC Ophthalmol. 2017 Sep 8;17:166. doi: 10.1186/s12886-017-0562-8 (PMC5591565; doi:10.1186/s12886-017-0562-8)
Supplement: Supplementary file 1 — 1. Searching strategy in pubmed for the comparison of ILM peeling group & non-ILM peeling group. 2. Searching strategy in pubmed for the comparison of Tamponade group & non-Tamponade group. (DOC 30 kb) [file 12886_2017_562_MOESM1_ESM.doc]

Additional file 1

1. Searching strategy in pubmed for the comparison of ILM peeling group & non-ILM peeling group.

free words

1=( near sight or nearsighted or myopia or shortsighted or short sight or myope or myopic defocus or HM) AND (macular retinoschisis or lamellar macular hole or MRS or LMH or foveoschisis) AND (internal limiting membrane peeling or ILMP)

truncations

2=( near sight or nearsighted* or myopia* or shortsighted* or short sight or myope* or myopic defocus or HM) AND (macular retinoschisis or lamellar macular hole or MRS or LMH or foveoschisis*) AND (internal limiting membrane peeling or ILMP)

MeSH

3=myopia AND retinoschisis AND (internal limiting membrane peeling or ILMP)

1 OR 2 OR 3

2. Searching strategy in pubmed for the comparison of Tamponade group & non-Tamponade group.

free words

1=( near sight or nearsighted or myopia or shortsighted or short sight or myope or myopic defocus or HM) AND (macular retinoschisis or lamellar macular hole or MRS or LMH or foveoschisis) AND (tamponade or fill or inflate)

truncations

2=( near sight or nearsighted* or myopia* or shortsighted* or short sight or myope* or myopic defocus or HM) AND (macular retinoschisis or lamellar macular hole or MRS or LMH or foveoschisis*) AND (tamponad* or fill* or inflat*)

MeSH

3=myopia AND retinoschisis AND (tamponade or fill or inflate)

1 OR 2 OR 3
