# Supplementary material for: Internal limiting membrane peeling and gas tamponade for myopic foveoschisis: a systematic review and meta-analysis
Source: BMC Ophthalmol. 2017 Sep 8;17:166. doi: 10.1186/s12886-017-0562-8 (PMC5591565; doi:10.1186/s12886-017-0562-8)
Supplement: Supplementary file 2 — 1. Outcome indicators of included studies in the comparison of ILM peeling group & non-ILM peeling group. MF, myopic foveoschisis; NR, not reported; Song et al.①, Song et al.②: two sets of data in the study of Song et al. 2. Outcome indicators of included studies in the comparison of Tamponade group & non-Tamponade group. MF, myopic foveoschisis; NR, not reported. (DOC 79 kb) [file 12886_2017_562_MOESM2_ESM.doc]

Additional file 2

1. Outcome indicators of included studies in the comparison of ILM peeling group & non-ILM peeling group.

|  | ILM Peeling | | | | |  | Non-ILM Peeling | | | |  |
| --- | --- | --- | --- | --- | --- | --- | --- | --- | --- | --- | --- |
| Author | Eyes | Resolution of MF(N) | Visual Acuity Improvement (N) | | Postoperative Complications (N) |  | Eyes | Resolution of MF(N) | Visual Acuity Improvement (N) | Postoperative Complications (N) |  |
| Li et al.[^22^](#_ENREF_22) | 4 | 0 | 1 | 0 | |  | 7 | 2 | 2 | 0 |  |
| Song et al.[^24^](#_ENREF_24)^①^ | 10 | 6 | NR | NR | |  | 24 | 16 | NR | NR |  |
| Song et al.[^24^](#_ENREF_24)^②^ | 13 | 10 | NR | NR | |  | 16 | 13 | NR | NR |  |
| Xu et al.[^26^](#_ENREF_26) | 14 | 12 | NR | 1 | |  | 15 | 10 | NR | 2 |  |
| Cai et al.[^21^](#_ENREF_21) | 14 | 10 | 3 | 0 | |  | 11 | 3 | 2 | 0 |  |
| Liu et al.[^23^](#_ENREF_23) | 16 | 11 | 9 | 0 | |  | 14 | 6 | 5 | 0 |  |
| Wang et al.[^25^](#_ENREF_25) | 15 | NR | 15 | NR | |  | 13 | NR | 13 | NR |  |
| MF, myopic foveoschisis; NR, not reported; Song et al.^①^, Song et al.^②^: two sets of data in the study of Song et al. | | | | | | | | | | | |

2. Outcome indicators of included studies in the comparison of Tamponade group & non-Tamponade group.

|  | Tamponade | | | | |  | Non-Tamponade | | | |  |
| --- | --- | --- | --- | --- | --- | --- | --- | --- | --- | --- | --- |
| Author | Eyes | Resolution of MF(N) | Visual Acuity Improvement (N) | | Postoperative Complications (N) |  | Eyes | Resolution of MF(N) | Visual Acuity Improvement (N) | Postoperative Complications (N) |  |
| Zhang et al.[^19^](#_ENREF_19) | 16 | 13 | 16 | 4 | |  | 24 | 16 | 24 | 0 |  |
| Song et al.[^24^](#_ENREF_24) | 13 | 10 | NR | NR | |  | 10 | 6 | NR | NR |  |
| Kim et al.[^8^](#_ENREF_8) | 9 | 8 | 5 | 2 | |  | 8 | 6 | 5 | 0 |  |
| Gui et al.[^20^](#_ENREF_20) | 15 | 10 | 15 | 0 | |  | 14 | 9 | 14 | 0 |  |
| MF, myopic foveoschisis; NR, not reported | | | | | | | | | | | |
