# Supplementary material for: Internal limiting membrane peeling and gas tamponade for myopic foveoschisis: a systematic review and meta-analysis
Source: BMC Ophthalmol. 2017 Sep 8;17:166. doi: 10.1186/s12886-017-0562-8 (PMC5591565; doi:10.1186/s12886-017-0562-8)
Supplement: Supplementary file 3 — a. Graph for risk of bias: All included studies are reviewed concerning the authors’ judgements on each bias risk item displayed as percentagesin the comparison of ILM peeling group & non-ILM peeling group. b. Summary for risk of bias: All included studies are reviewed concerning the authors’ judgements on each bias risk item in the comparison of ILM peeling group & non-ILM peeling group. Figure S2. a. Graph for risk of bias: All included studies are reviewed concerning the authors’ judgements on each bias risk itemdisplayed as percentages in the comparison of Tamponade group & non-Tamponade group. b. Summary for risk of bias: All included studies are reviewed concerning the authors’ judgements on each bias risk item in the comparison of Tamponade group & non-Tamponade group. (DOCX 254 kb) [file 12886_2017_562_MOESM3_ESM.docx]

Additional file 3


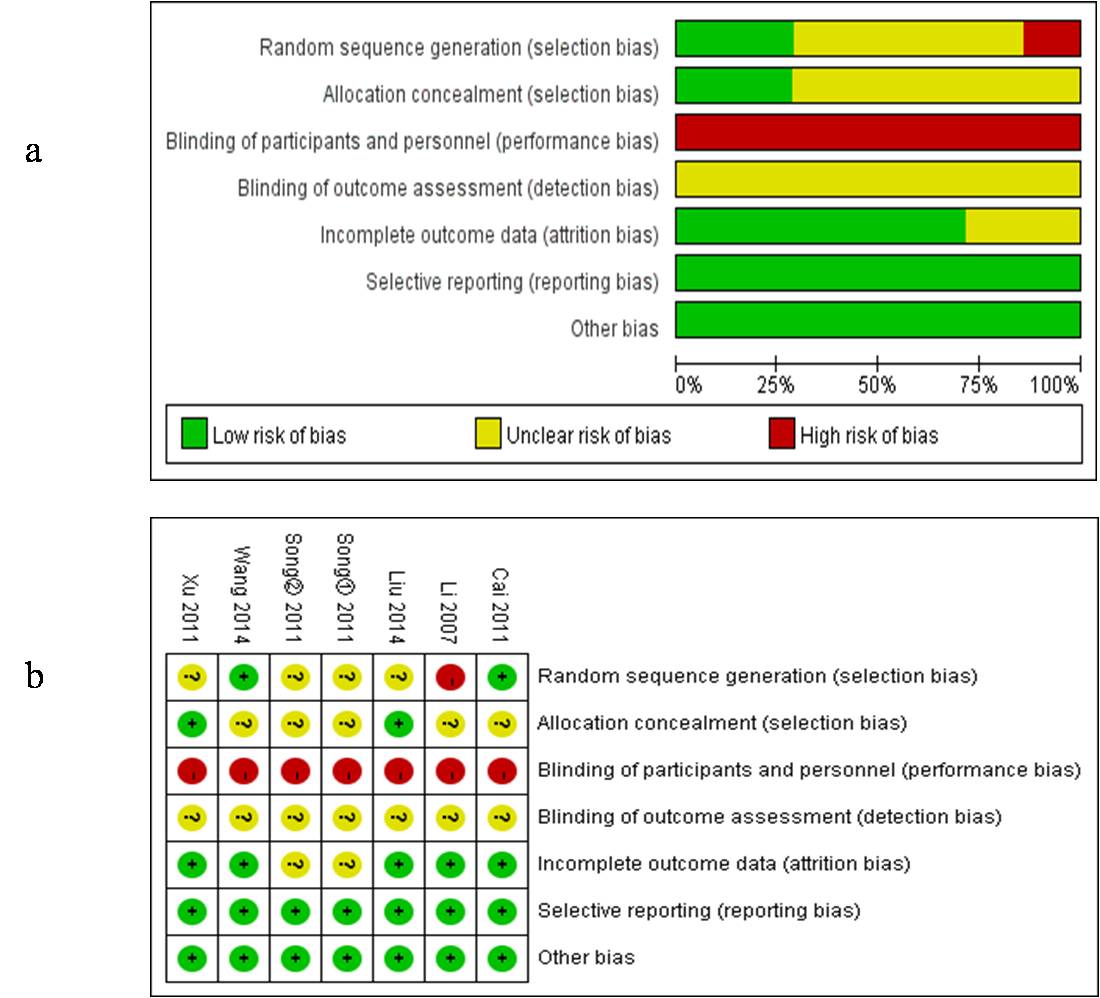


Figure 1

a. Graph for risk of bias:

All included studies are reviewed concerning the authors’ judgements on each bias risk item displayed as percentagesin the comparison of ILM peeling group & non-ILM peeling group.

b. Summary for risk of bias:

All included studies are reviewed concerning the authors’ judgements on each bias risk item in the comparison of ILM peeling group & non-ILM peeling group.


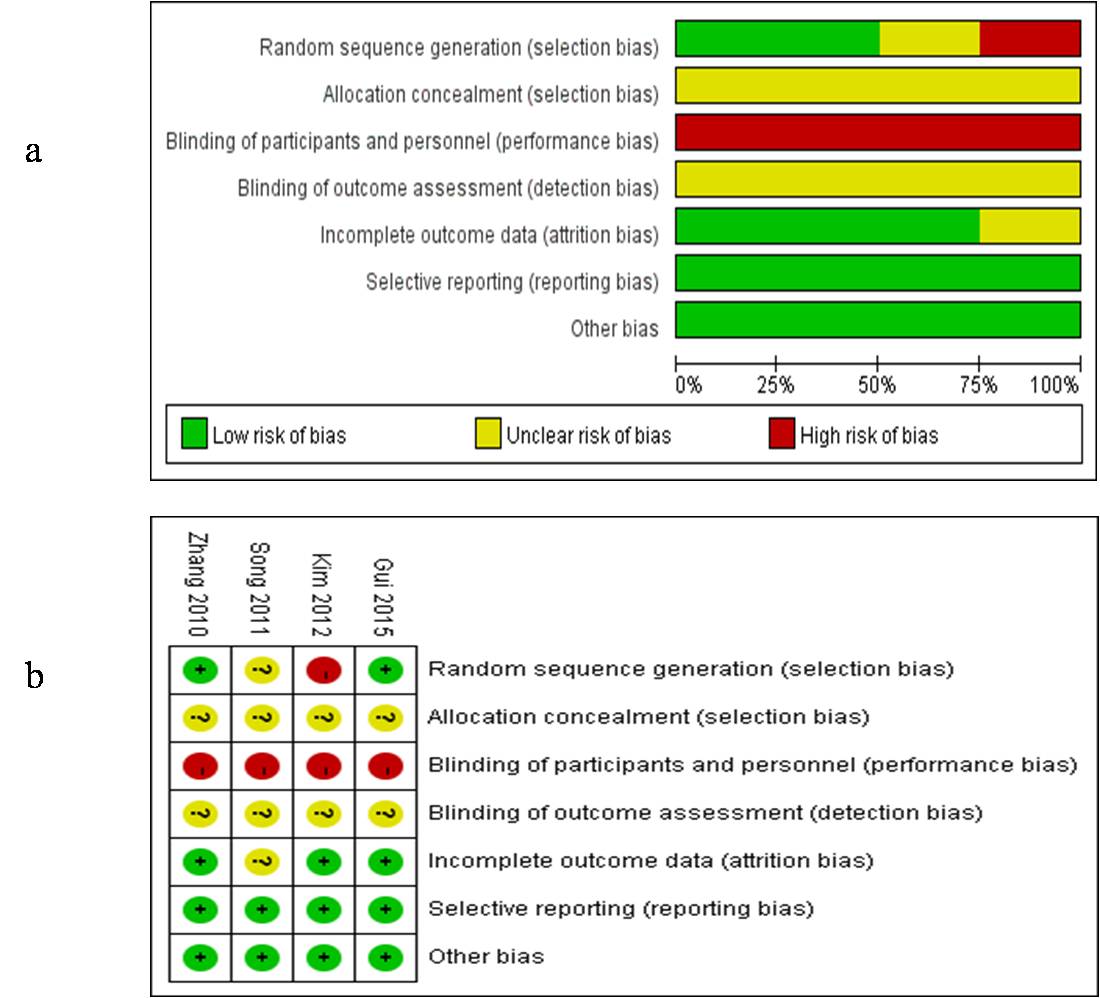


Figure 2

a. Graph for risk of bias:

All included studies are reviewed concerning the authors’ judgements on each bias risk item displayed as percentages in the comparison of Tamponade group & non-Tamponade group.

b. Summary for risk of bias:

All included studies are reviewed concerning the authors’ judgements on each bias risk item in the comparison of Tamponade group & non-Tamponade group.
